# Supplementary material for: Overexpression of Heat Shock Protein 70 Improves Cardiac Remodeling and Survival in Protein Phosphatase 2A-Expressing Transgenic Mice with Chronic Heart Failure
Source: Cells. 2021 Nov 15;10(11):3180. doi: 10.3390/cells10113180 (PMC8624068; doi:10.3390/cells10113180)
Supplement: Supplementary file 1 [file cells-10-03180-s001.zip › cells-1375624-supplementary.pdf]

cells MDPI

Supplementary Materials:

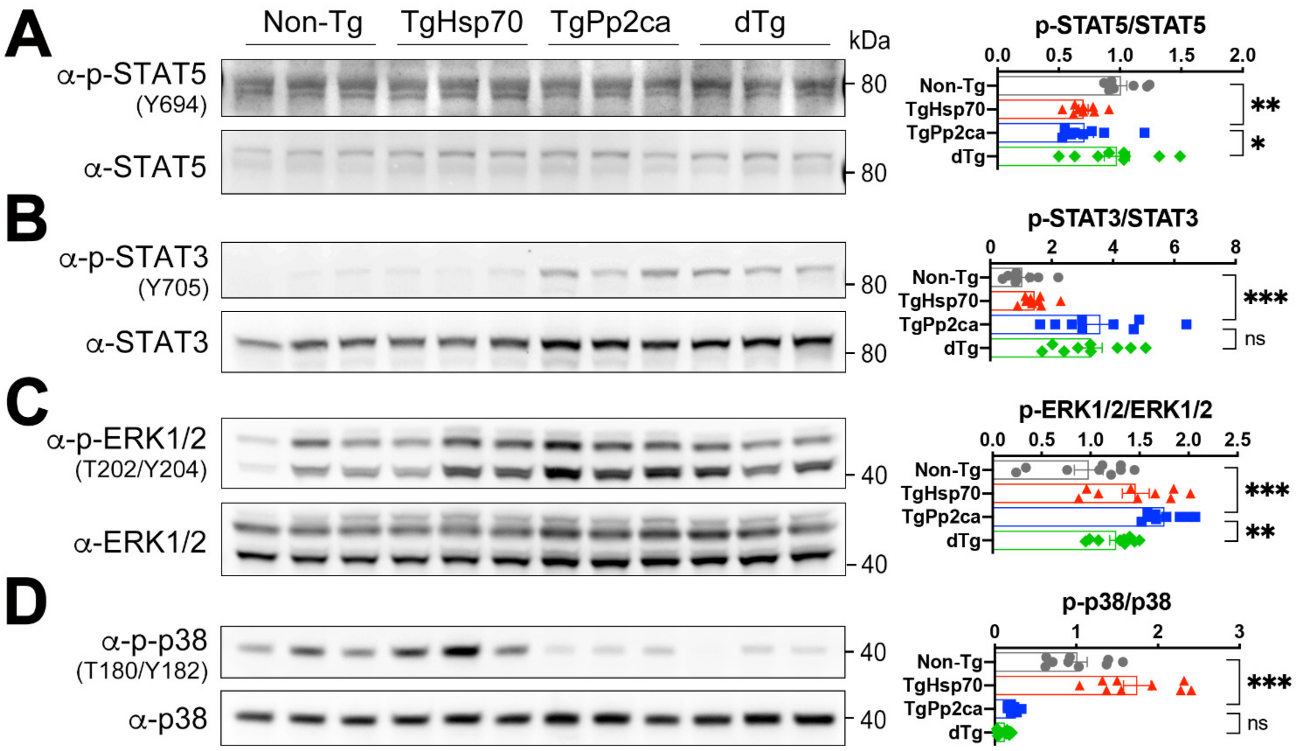

Figure S1. Phosphorylation changes in hearts from mice at 6 months of age. (**A**) Phosphorylated signal transducer and activator of transcription (STAT) 5 (Tyr694) (**B**) Phosphorylation of STAT3 (Tyr705) (**C**) Phosphorylation of extracellular signal-regulated kinase 1/2 (Thr202/Tyr204) (ERK1/2) (**D**) Phosphorylated p38 (Thr180/Tyr182). Dot blot in the right side of the western blot mean densitometer analysis. One way ANOVA and LSD post hoc was applied. *, $p < 0.05$; **, $p < 0.01$; ***, $p < 0.001$; NS, not significant.

.

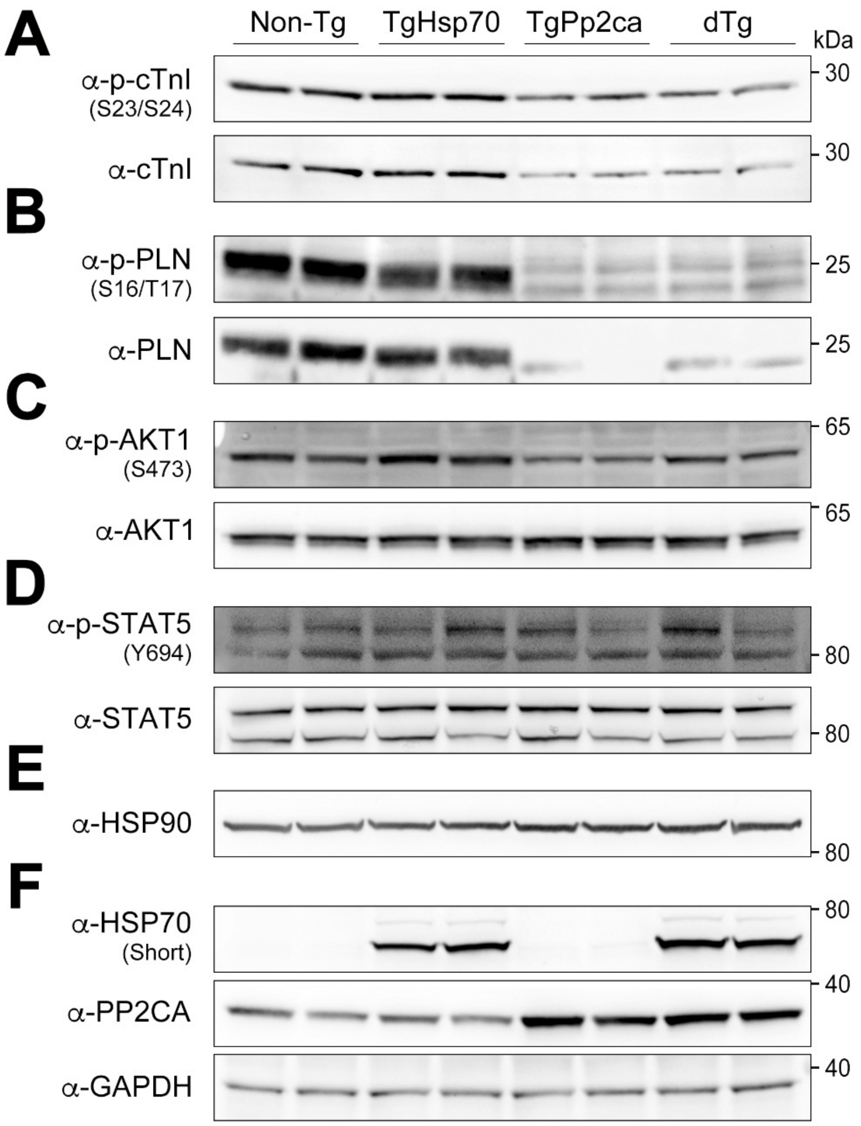

Figure S2. Phosphorylation profiles in hearts from younger mice. Phosphorylation changes in hearts of 7-week-old mice were assessed. Phosphorylation of cardiac troponin I (Ser23/24) (**A**), phospholamban (Ser16/Thr17) (**B**), and phosphorylation levels of protein kinase B (Ser473) (AKT1, **C**). Phosphorylation of AKT was reduced in TgPp2ca mice, and expression of HSP70 ameliorated Pp2ca-mediated dephospohrylation of AKT. (**D**) Phosphorylation of STAT5 (Tyr694). (**E**) HSP90 expression was not affected. (**F**) Expression of each protein represents transgenic overexpression of each protein. .
